# Snakes and snakebite envenoming in Northern Tanzania: a neglected tropical health problem

**DOI:** 10.1186/s40409-015-0033-8

**Published:** 2015-08-26

**Authors:** M. J. Kipanyula, W. H. Kimaro

**Affiliations:** Department of Veterinary Anatomy, Faculty of Veterinary Medicine, Sokoine University of Agriculture, Chuo Kikuu, PO Box 3016, Morogoro, Tanzania

**Keywords:** Venomous snakes, Snakebites, Antivenom, Meserani, Tanzania

## Abstract

**Background:**

Snakebites cause considerable human and livestock injuries as well as deaths worldwide, and particularly have a high impact in sub-Saharan Africa. Generating a basic platform of information on the characteristics of snakes and snakebites in various countries is relevant for designing and implementing public health interventions.

**Methods:**

This study was performed to identify types of snakes and some of the characteristics of snakebite cases in two communities, an agricultural and a pastoralist, in Arusha region, northern Tanzania. A total of 30 field visits were carried out in areas considered by local inhabitants to be potential microhabitats for snakes. Direct observation of snake types based on morphological features and a structured questionnaire were employed for data collection.

**Results:**

A total of 25 live and 14 dead snakes were encountered. Among the dead ones, the following species were identified: two black-necked spitting cobras (*Naja nigricollis*); five puff adders (*Bitis arietans*), one common egg-eater (*Dasypeltis scabra*); two rufous-beaked snakes (*Ramphiophis rostratus*); two brown house snakes (*Lamprophis fuliginosus*); one Kenyan sand boa (*Eryx colubrinus*), and one black mamba (*Dendroaspis polylepis*). The frequency of snake encounters was significantly higher (*χ*^2^ = 4.6; *p* = 0.03) in the pastoral than in the agricultural area; there were more snakebite cases in the former, but the differences were not statistically significant (*p* = 0.7). A total of 242 snakebite victims attended at the Meserani Clinic, located in the study area, between the years 2007 to 2012. Of all cases, 146 (61.6 %) and 96 (38.4 %) were male and female patients, respectively. As for age distribution, 59.1 % of snakebite victims were from the economically active age groups between 15 and 55 years.

**Conclusion:**

Snakebites are a threat to rural communities and public health in general. The burden of snakebites in Tanzania presents an epidemiologically similar picture to other tropical countries. Livestock keeping and agriculture are the major economic activities associated with snakebites. Community-based public education is required to create awareness on venomous snakes and predisposing factors to snakebites. These tasks demand integration of diverse stakeholders to achieve a common goal of reducing the impact of human suffering from these envenomings in Tanzania.

## Background

Snakebite envenomings constitute a neglected tropical disease of high impact on a global basis, disproportionately affecting impoverished populations in rural settings of Africa, Asia and Latin America [1–5]. It has been estimated that the annual load of envenomings worldwide is above two million cases, provoking more than 100,000 deaths and leaving about 400,000 people with permanent sequelae [1, 6, 7]. In sub-Saharan Africa, these envenomings have a serious impact in terms of morbidity and mortality owing to factors such as abundance of snakes, subsistence agricultural and pastoralist activities, poor availability and accessibility of antivenoms, and inadequately trained health workers for attending this medical emergency, among other factors [8, 9]. A consequence of this scenario is that many people suffering snakebite do not attend health facilities and instead, rely on local traditional healers, a fact that further complicates this problem [10].

Tanzania, being in the eastern sub-Saharan African region, presents a rich variety of venomous snakes [11, 12]. According to the WHO [12], the species having the highest medical impact in this country are the spitting cobras *Naja nigricollis* and *Naja mossambica*, the neurotoxic mambas *Dendroaspis angusticeps* and *Dendroaspis polylepis*, of the family Elapidae and the puff adder, *Bitis arietans*, of the family Viperidae. In addition, other species of the families Elapidae, Viperidae and Colubridae provoke many cases and induce severe envenomings in this country [12, 13]. Despite the relevance of this public health problem, there is very little published information on the impact of this tropical disease in Tanzania.

The present study aimed to provide preliminary information on several aspects of the snakebite problem in two different settings associated with agricultural and pastoralist activities in Arusha region, northern Tanzania. Specifically, the study investigated the predominant types of snakes in the region, and approximated the frequency of snakebites in the study areas, as well as socioeconomic factors predisposing the community members to snakebites; likewise, the basic demographic features of snakebitten patients in a clinic were studied.

## Methods

### Study area

**Fig. 1:**
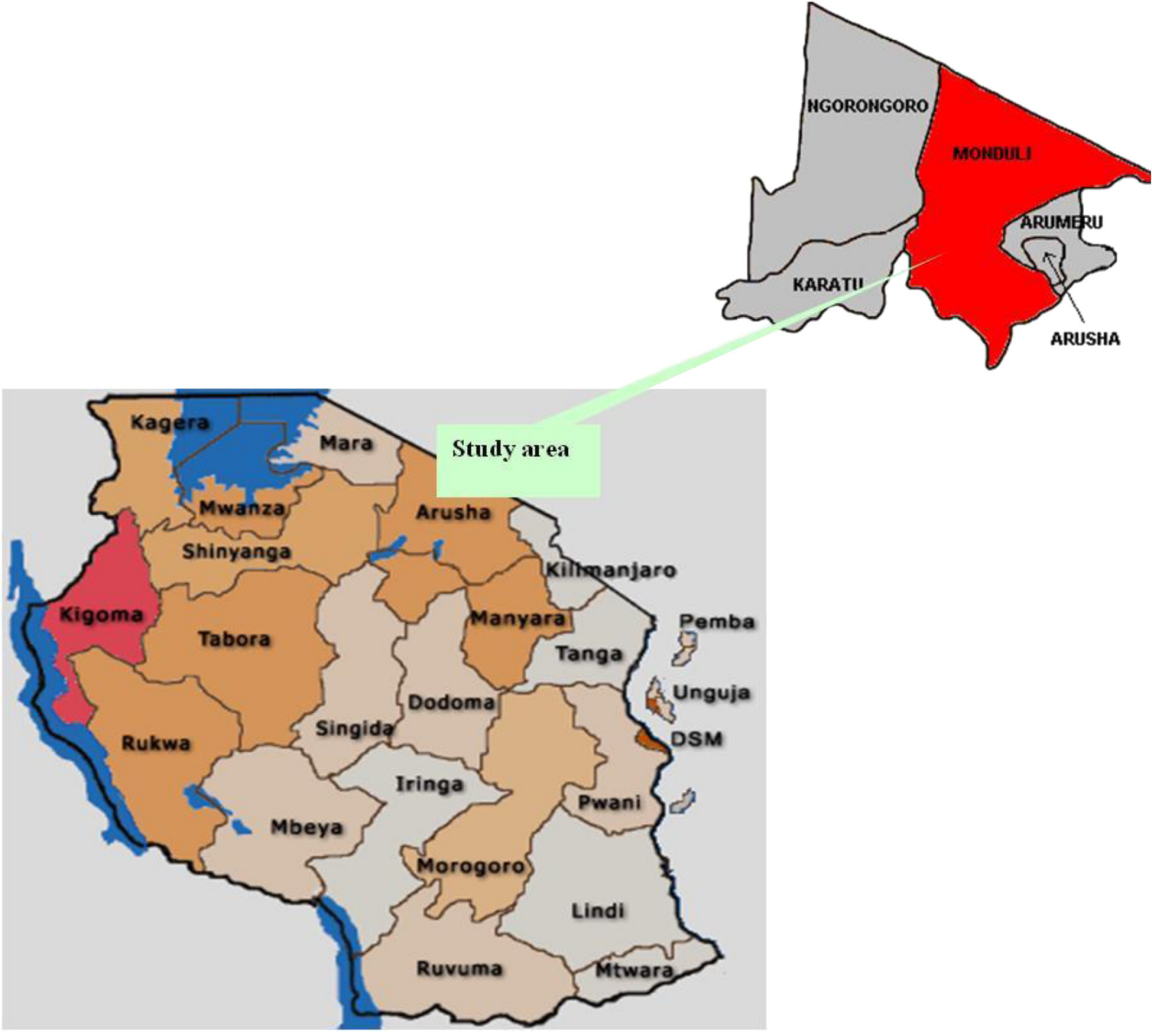
Map of the United Republic of Tanzania showing adminstrative regions, as well as Monduli district (*in red*) and the neighboring Arumeru district, in Arusha, where the two communities investigated in this study are located (image adapted from: http://en.wikipedia.org/wiki/Arusha_Region)

The study was conducted in two villages, namely Meserani Juu and Lesiraa, in the Monduli and Arumeru districts, respectively, in Arusha region, Tanzania. The districts are 1534 m above sea level between latitude 3°17′59″ S and longitude 36°27′00″ E (Fig. 1). Agriculture is the main economic activity practiced by community members in Meserani Juu village. In contrast, Lesiraa village is open grassland used by pastoralists (Maasai communities) for cattle grazing. The village is situated in a semiarid area characterized by a prolonged dry season lasting up to 7 months. The minimum and maximum average ambient temperatures are 16 and 27 °C, respectively. Selection of the study sites was based on the fact that the areas present microhabitats whose ecological features are associated with a rich herpetofauna and thus highly prone to reports of snakebite cases.

### Data collection

#### Direct observation and on site identification of snakes

The study way carried out for 1 month (30 days) from January to February of 2013. Field visits were conducted for the 30-day study period and every visit involved a different area to identify live and dead snakes around the selected villages. A total of 30 visits were completed, comprising 15 visits to each of the study areas. A visual encounter survey method was employed for snake collection and study areas were visited on a regular basis. All snake species observed were recorded and dead snakes were collected and preserved in 10 % formalin for further identification.

During regular surveys, searching was conducted in all possible areas considered by local inhabitants to be potential microhabitats for snakes. Target areas included the sunny side of small rock area, rock walls, fallen logs near roads, pockets of leaves, margins of streams, agricultural fields, forest, bushes and human settlements during daytime. Direct morphological observations of snakes encountered were based on their characteristic anatomical features, and were performed by a researcher trained specifically for this work. For the identification of snakes, reference was made to Spawls et al. [14], and Cobom [15], and where dead snakes were encountered; a herpetologist (Casian Mluge, Department of Veterinary Anatomy, Sokoine University of Agriculture) was consulted for identification. On average each of the field visits covered a territory of about 9–25 km^2^. Survey time was typically from 7 to 11 h and from 16 to 19 h, whereas searches were generally avoided during midday because of reduced snake activity during that time. Night surveys were avoided due to safety reasons.

### Questionnaire for collecting information on the local population

The quantitative part of the study was conducted in the community whereby the households were randomly selected in all three areas. Primary data were collected through a structured questionnaire. Purposive sampling and simple random sampling was used to select sample size in each village. To avoid repetition of data, one questionnaire was administered to one participant from each household. Only individuals older than 18 years with a minimum of 3 years stay in the village were interviewed. Gender was considered so that at least 50 % of the respondents were women.

Participation was on a voluntary basis and oral consent was sought before the interview. The questionnaire was comprised of both closed- and open-ended questions. Open-ended questions were employed to provide the respondents a chance to explain further on issues asked. This technique provided valuable information which showed different environments and circumstances where humans encountered snakes in their daily life. The following main issues were addressed in the questionnaire: (a) frequency of encounters with snakes; (b) frequency of snakebites; (c) knowledge that people have on snakes; (d) views and conceptions that people have on snakes. The response after snake encounters was also investigated. Different social and economic activities that expose human beings to snakebite as well as seasons with high snakes encounters were also studied.

#### Information on snakebite cases

Data on snakebites were collected from the Meserani Clinic located at the Meserani Snake Park, Arusha, northern Tanzania. This clinic is the only place where antivenom is readily available in northern Tanzania [16]. A checklist of information to be recorded was prepared and used to collect data on snakebites. Information on snakebites included: the number of snakebite cases managed by the clinic, the age of the victims, as well as the type of treatment and antivenom used.

### Data analysis

All the raw data were entered on Excel 2010 spreadsheets (Microsoft Corporation, USA). Statistical Package for the Social Sciences (SPSS) version 16.0 was used to generate the frequencies (%) of different variables from which the tables were then generated. Chi-square (*χ*^2^) test at *p* < 0.05 was considered statistically significant for the tested hypotheses.

### Ethics committee approval

Permission to carry out this study was granted by district directors of respective areas and ethics approval for the study was given by the Ethical Committee of Sokoine University of Agriculture (SUA), Morogoro, Tanzania. The Vice Chancellor of SUA issued a research permit letter on behalf of the Tanzanian Commission for Science and Technology (COSTECH) that permitted the researchers to carry out the study. Verbal consent was obtained from each respondent after explaining the purpose and importance of the study prior to data collection. The respondents were assured of their right to withdraw from the interview at any time they would wish during the discussion.

## Results

### Field visits and identification of snakes

At total of 30 field visits were made comprising of 15 visits to each of the two study areas. During field visits, a total of 39 snakes were encountered. Twenty-five live snakes were viewed by the researchers in the field, and 14 dead snakes were found. Dead snakes were collected and referred to the herpetologist for identification based on gross anatomical features and morphological characteristics. The 14 dead snakes sent for identification belonged to four families (Elapidae, Viperidae, Colubridae and Boidae), as shown in Table 1. The following snakes were identified by the herpetologist: two black-necked spitting cobras (*Naja nigricollis,* family Elapidae); five puff adders (*Bitis arietans,* family Viperidae), one common egg eater (*Dasypeltis scabra,* family Colubridae)*;* two rufous-beaked snakes (*Ramphiophis rostratus,* family Colubridae); two brown house snakes (*Lamprophis fuliginosus,* family Colubridae), one Kenyan sand boa (*Eryx colubrinus,* family Boidae*)* and one black mamba (*Dendroaspis polylepis,* family Elapidae) (Table 1).

**Table 1:** List of snake species recorded in Meserani Juu village (Monduli district) and Lesiraa village (Arumeru district) in the Arusha region. The list is based on dead snakes collected in field and identified by a specialist

Scientific name	Common name	Family	Number of specimens identified	Venomous or non-venomous
*Bitis arietans*	Puff adder	Viperidae	5	Venomous
*Naja nigricollis*	Black-necked spitting cobra	Elapidae	2	Venomous
*Dasypeltis scabra*	Common egg eater	Colubridae	1	Non-venomous
*Ramphiophis rostratus*	Rufous-beaked snake	Colubridae	2	Venomous^a ^
*Eryx colubrinus*	Kenyan sand boa	Boidae	1	Non-venomous
*Dendroaspis polylepis*	Black mamba	Elapidae	1	Venomous
*Lamprophis fuliginosus*	Brown house snake	Colubridae	2	Non-venomous

^a^This colubrid species possesses venom-secreting glands, but due to the disposition of fangs (opisthoglyphous), bites associated with envenomings in humans are unlikely

### Snakes encounters by community members

We interviewed 75 respondents living in the two communities (40 in Mesarani Juu and 35 in Lesiraa) to gather information about snake encounters in the study area. Participation was on a voluntary basis following oral consent for the interview. Respondents were free to withdraw from the interview at any point. Ninety-two percent of responders indicated that they had seen snakes in their village. The majority of respondents in both locations had seen a snake more than twice in 1 year. The frequency of snake encounters, i.e. having had a close contact with a snake, was significantly higher (*χ*^2^ = 4.6; *p* = 0.03) in the pastoralist area, where 74 % of respondents reported such encounters, versus only 50 % in the agricultural region. Many community members encountered snakes during the daytime, with 58 (77.2 %) of the respondents indicating that they normally spot snakes during midday hours on roads, surfaces of rocks, logs, grasses and many other places that expose snakes to sunlight. Community members encountered snakes in agricultural areas particularly during short rainy seasons while clearing fields for cultivation, and during the dry season, when harvesting crops and in grazing areas during livestock herding. Based on the color and appearance of snakes, 52 % of all respondents to the questionnaire indicated that they normally encounter black snakes, 29.3 % reported having seen green snakes, 5.3 % indicated familiarity with purple snakes, 2.7 % indicated having observed brown snakes and 10.7 % appeared to be familiar with snakes of other colors.

Activities that exposed community members to snakes and snakebite were agriculture, herding, hunting and working on development sites. Twenty-nine of the respondents (38.7 %) encountered snakes in agriculture areas, 34 respondents (45.3 %) in grazing areas, and 12 respondents (16 %) in other locations.

From the information gathered, it is concluded that snakebites are frequent at both locations studied. Data collected at community level indicated that five out of 35 interviewed people (14.3 %) were bitten by snakes in Lesiraa (pastoralist region), and three out of 40 people (7.5 %) were bitten in Meserani Juu (agricultural region) in the past 5 years. These differences were not statistically significant (*p* > 0.7).

### Inhabitants’ knowledge on snakes

Typically, persons fear snakes owing to the possibility that their lives may be in danger upon encounter, although this perception varies on an individual basis. Out of 75 respondents interviewed in this study, 37 (49.3 %) indicated being terrified upon encountering a snake, whereas 27 respondents (36 %) reported feeling excitement, while 11 respondents (14.7 %) were indifferent to this event. Interestingly, 92 % of the respondents indicated that the snakes usually fled upon an encounter with humans. As to their knowledge on venomous snakes, 77.1 % and 85 % of respondents in Lesiraa and Meserani Juu villages, respectively, believed that all snakes are venomous and that it is why they killed them upon encounter; this difference between communities was not statistically significant (*p* > 0.05).

Most of the respondents could not precisely differentiate between venomous and non-venomous snakes. Out of those who said they were able to distinguish the two, 77.1 and 45 % of the respective respondents in Lesiraa and Meserani Juu villages, used color and shape of the head to differentiate venomous from non-venomous snakes. Respondents’ descriptions based on anatomical and morphological features and color of encountered snakes suggested that the black-necked spitting cobra, black mamba*,* eastern green mamba, puff adder, brown house snake, mole snake and rufous-beaked snake were the most frequently encountered snakes. On the basis of their ability to distinguish between venomous and non-venomous snakes, it was observed that repondents in Lesiraa village were more knowledgeable than those in Maserani Juu village, although the differences were not statistically significant. Some respondents in the pastoralist area indicated that the most dangerous snake was the one that spreads its hood or raises a flattened neck, features characteristic of cobras (genus *Naja*, family Elapidae). In addition, many interviewees responded that the venomous snake was the one with spade head—identifying viperid species, i.e. puff adder (*Bitis arietans*).

### The burden of snakebites in a clinic

The number of human snakebites cases reported at the Meserani Clinic, Arusha, is presented in Table 2. A total of 242 suspected snakebite cases were recorded between the years 2007 to 2012. Of all cases, 146 (61.6 %) and 96 (38.4 %) were male and female patients, respectively. For the year 2012, the age group distribution of the reported snakebite cases indicated that 59.1 % of victims were between 26 and 55 years, 25 % from 18 to 25 years, 8.4 % above 56 while 7.2 % were between 0 and 17 years (Table 3). Some of the snakebite victims were treated by using polyvalent antivenom (ASV) at the Meserani Clinic.

**Table 2:** Number of snakebite cases reported at the Meserani Clinic, Arusha in the period 2007 to 2012

Year	Number of snakebite cases reported at Meserani clinic
2007	5
2008	35
2009	50
2010	49
2011	47
2012	56
Total	242

**Table 3:** Age group distribution of snakebite cases reported at Meserani clinic, Arusha, in 2012

	Age group of snakebite victims	Number
	0–10	7
	11–20	15
	21–30	14
	31–40	7
	41–50	9
	51–60	4
Total		56

## Discussion

Snakebite envenomings constitute a significant public health threat in sub-Saharan Africa [3, 6, 17]. Nevertheless, this disease is severely neglected by public health authorities and non-governmental organizations [3, 18]. One of the reasons behind such wide neglect is the lack of information on the magnitude of the problem and on its medical, social and economic implications for agricultural and pastoralist communities in the region. Thus, it is necessary to develop investigations on several aspects of snakebite envenoming in sub-Saharan African countries, with special emphasis on studies at the community level, in order to gain novel information on the particular aspects of this pathology in various cultural and economic contexts in the region.

One of the first goals of our study was to identify the most prevalent snakes in two regions of Tanzania, i.e. Lesiraa and Meserani Juu, inhabited by pastoralist and agricultural communities, respectively. Field observations of snakes collected in the field allowed the identification of several species of venomous snakes, such as the black-necked spitting cobra (*Naja nigricollis*) and the black mamba (*Dendroaspis polylepis*) of the family Elapidae, and the puff adder (*Bitis arietans*) of the family Viperidae. Coincidentally, these three species are listed in category 1 in Tanzania by the World Health Organization, which corresponds to species of “highly venomous snakes which are common or widespread and cause numerous snakebites, resulting in high levels of morbidity, disability or mortality” [12, 13]. In addition, a number of snakes from the families Colubridae and Boidae were also identified. A similar list of snakes was identified in the Dodoma region further suggesting that these are likely to be the most common species of snakes found in Tanzania [19]. Moreover, a study of patients attended at the Meserani clinic in Arusha, northern Tanzania, revealed that these snake species were responsible for the majority of cases received in this health center [16].

The impact and cultural perceptions on snakebites were examined in two different communities devoted to agricultural and pastoralist activities, in order to gain further insights on this disease at the community level, and to assess whether lifestyle differences may have implications on snakebites. Our observations indicated that 92 % of the respondents had observed a snake, stressing the risk of snakebites in this population. Results also indicate that 72.2 and 50 % of the interviewees in the pastoralist and agricultural communities, respectively, had had a close encounter with a snake. Moreover, in the last 5 years, 14.3 and 7.5 % of interviewed people have suffered at least one snakebite in Lesiraa (pastoralist community) and Meserani Juu (agricultural community), respectively, further indicating that snakebite is a frequent hazard in the life of these communities. Although differences in the number of snakebite cases recorded between pastoralist and agricultural communities were not statistically significant, the former tended to be higher. The trend observed is in agreement with data reported elsewhere where a high burden of snakes in pastoralist areas in Iran was attributed to presence of an ideal habitat for snakes, as compared to agricultural areas, although snakebite often occurs in both types of regions [20]. Certainly, human activities can affect the frequencies human-snake encounters.

Members of both communities had a strong feeling of fear towards snakes. Typically, members of rural communities fear snakes for the possibility that their lives may be in danger upon the encounter [21, 22]. Many pastoralists and farmers interviewed consider all snakes to be venomous and dangerous regardless of their species, which is the reason for killing them upon encounter. A relatively low percentage of interviewees could differentiate between venomous and non-venomous snakes. Based on how respondents were able to describe and distinguish venomous from non-venomous snakes based on morphological features and color, it was apparent that people living in the pastoralist community had relatively more knoweledgeable on snakes than people living in the agricultural community. Our observations stress the need to develop community-based programs for improving the local knowledge on snakes and snakebites, and especially to promote basic preventive measures at the community level to reduce the incidence of snakebites cases. It is also necessary to perform additional community-based studies aimed at identifying the main human risk factors exposing community members to snakes and snakebites in both agricultural and pastoralist areas, in order to design appropriate preventive interventions tailored to the specific contexts in these communities. The design and implementation of these programs should avoid a top-down approach but rather should actively incorporate local community members in the planning and performance of these activities [23].

Information on snakebites was also collected at the Meserani clinic in Arusha, northern Tanzania, a local clinic where snake bitten patients are attended and antivenom is provided free of charge [16]. In a 5-year period, this clinic received 242 snakebite victims. The majority of snakebite victims were between 15 and 55 years old, hence being in the economically active age groups, although cases of children less than 10 years of age and adults older than 60 years were also reported. Similar age groups affected by snakebites have been reported elsewhere in Africa [24–26]. This highlights one of the main aspects of snakebite envenoming on a worldwide basis, i.e. it largely affects economically productive persons in rural communities [3, 18]. This, in turn, has profound implications from a biosocial perspective that include not only provoking biomedical consequences, such as morbidity and mortality, but also generating social suffering on a wider scale. Death, permanent disability or prolonged absence from work will bring serious social and economic consequences to families and communities, an issue that has received little attention from research agendas and public health authorities, and must be documented through community-based surveys.

Our study only allowed the collection of basic information on snakebites in a region of Tanzania. It is necessary to further investigate the main clinical features and the most common complications of these envenomings. A previous study revealed the existence of two main clinical presentations, i.e. envenomings associated with local tissue damage caused with *B. arietans* and cytotoxic cobras (*N. nigricollis*) bites, and envenomings associated with neurotoxic manifestations provoked by neurotoxic cobras (*Naja* sp.) and mambas (*Dendroaspis* sp.) [16]. Meserani clinic, where data on snakebitten patients were collected, is the only place where antivenoms are readily and freely available in northern Tanzania [16]. The antivenom being used in this clinic is a polyspecific product manufactured by the South African Medical Research Institute (SAIMR), and has proven effective in the treatment of envenomings in Tanzania [16]. Besides this initiative, access to antivenom is difficult and inconsistent in this country, as occurs in most of sub-Saharan Africa [9, 10]. This issue of poor antivenom availability and accessibility in this region of the world is a serious and complex one having multifactorial causes [9–11, 27]. Its solutions demand strong advocacy and effective actions at various levels, both national and international [17, 23].

As other antivenoms are being manufactured for sub-Saharan Africa, using variable immunization schemes with different venom mixtures, it is necessary to evaluate, at the preclinical level, the efficacy of these antivenoms against snake venoms from Tanzania, using conventional laboratory-based methodologies [13, 28]. For this, it is first necessary to obtain samples of venoms of the most medically relevant venoms in the country, such as *N. nigricollis*, *D. polylepis* and *B. arietans*. Hence, efforts are needed in order to generate high-quality pools of freeze-dried venoms from snakes of Tanzania, and to use them for testing various antivenoms available for distribution in this country. Based on these results, the public health authorities in the country should develop antivenom procurement and distribution programs in order to bring antivenoms to the health clinics in regions of high incidence of snakebites. In addition, the training of health staff in the diagnosis and appropriate management of snakebite envenomings is of paramount relevance in order to ensure effective treatment of envenomings and their complications.

## Conclusions

Snakebites are a threat to rural communities and public health in general. Identification of the most common snake species found in a particular area is crucial for proper management of snakebite cases in both humans and livestock. The burden of snakebites in Tanzania presents a picture epidemiologically similar to other tropical countries. Herding and agriculture are the major economic activities associated with snakebites. In order to deal with the snakebite problem in both humans and other animals, community-based public education is required to raise awareness on venomous snakes and predisposing factors to snakebites. For this, it is necessary to gather information on the situation and cultural perceptions of the problem in diverse rural settings, such as the ones explored in the present study. This will enable communities to develop effective strategies for enhancing both the prevention of snakebites and the conservation of snakes as wildlife resources. In addition to preventive campaigns at the community level, interdisciplinary programs aimed at generating a robust epidemiological database of snakebites, preclinical assessment of the efficacy of existing antivenoms, programs for the effective procurement and distribution of antivenoms, and improvement of the clinical management of envenomings by health staff are urgently required. These tasks demand the integration of diverse stakeholders, involving the public and private sectors, to achieve the common goal of reducing the impact of human suffering induced by these envenomings in Tanzania.
